# Determining Diagnostic Criteria of Unexplained Recurrent Implantation Failure: A Retrospective Study of Two *vs* Three or More Implantation Failure

**DOI:** 10.3389/fendo.2021.619437

**Published:** 2021-07-22

**Authors:** Yingying Sun, Yile Zhang, Xueshan Ma, Weitong Jia, Yingchun Su

**Affiliations:** ^1^ Center for Reproductive Medicine, The First Affiliated Hospital of Zhengzhou University, Zhengzhou, China; ^2^ Henan Key Laboratory of Reproduction and Genetics, The First Affiliated Hospital of Zhengzhou University, Zhengzhou, China; ^3^ Henan Provincial Obstetrical and Gynecological Diseases (Reproductive Medicine) Clinical Research Center, The First Affiliated Hospital of Zhengzhou University, Zhengzhou, China

**Keywords:** outcome, embryos transfer cycles, factor, definition, recurrent implantation failure

## Abstract

**Background:**

The definition of recurrent implantation failure (RIF) differs clinically, one of the most controversial diagnostic criteria is the number of failed treatment cycles. We tried to investigate whether the two implantation failure could be included in the diagnostic criteria of RIF.

**Methods:**

A retrospective analysis of the clinical data of patients (N=1518) aged under 40 years with two or more implantation failure, recruited from the Center for Reproductive Medicine of the First Affiliated Hospital of Zhengzhou University from January 2016 to June 2019.

**Results:**

After adjusting for confounding factors by using binary logistic regression, the results showed that partial general information and: distribution of associated factors were significant differences such as maternal age (*aOR*=1.054, *P*=0.001), type of cycle (*aOR*=2.040, *P*<0.001), stage of embryos development (*aOR*=0.287, *P*<0.001), number of embryos transferred (*aOR*=0.184, *P*<0.001), female factor (tubal pathology) (*aOR*=0.432, *P*=0.031) and male factor (*aOR*=1.734, *P*=0.002) between the groups with two and three or more unexplained implantation failure. And further explored whether these differential factors had a significant negative impact on pregnancy outcome, the results showed that: for patients who had three unexplained implantation failure, in the fourth cycle of ET, the live birth rate decreased significantly with age (*aOR*=0.921, *P*<0.001), and the live birth rate of blastocyst transfer was significantly higher than that of cleavage embryo transfer (*aOR*=1.826, *P*=0.007). At their first assisted pregnancy treatment after the diagnosis of RIF according to these two different definitions, there were no significant difference in the biochemical pregnancy rate, clinical pregnancy rate, ectopic pregnancy rate and abortion rate (*P*>0.05), but the live birth rate (35.64% *vs* 42.95%, *P*=0.004) was significantly different. According to the definition of ‘two or more failed treatment cycles’, the live birth rate of the first ET treatment after RIF diagnosis was significantly lower than that of patients according to the definition of ‘three or more failed treatment cycles’.

**Conclusion:**

For patients with unexplained recurrent implantation failure, two implantation failure cannot be included in the diagnostic criteria of RIF. This study supports the generally accepted definition of three or more failed treatment cycles for RIF.

## Introduction

The implantation rate per embryo transfer in assisted reproductive technology (ART) is approximately 30%, while the incidence of recurrent implantation failure (RIF) (universally applied definition is ‘three or more failed treatment cycles’) *in vitro* fertilization (IVF) patients is as high as 10% (1, 2). RIF is still the most challenging clinical dilemma because the overall clinical pregnancy rate of IVF in patients with RIF is extremely low. However, the definition of RIF differs clinically, and the most controversial diagnostic criteria include the number of failed treatment cycles, the number of embryos transferred and the maternal age (3, 4). In terms of the debate over the number of failed treatment cycles in definition for RIF, the generally accepted view is ‘three or more failed treatment cycles’ (1), but there are also many studies state that ‘two consecutive failed treatment cycles’ are sufficient to evaluate the occurrence of RIF (3, 5–7).

The hinge of successful embryo implantation dependents on the high quality of embryo and endometrial receptivity. With the advancement of ART, about 70% of transferred embryos have been identified as high-quality embryos recently (8), it seems that the failure of embryo implantation failure is more closely related to endometrial receptivity. A recent literature showed that patients with unexplained RIF, GnRH agonist combined with letrozole could significantly improve endometrial receptivity, thus increasing the clinical pregnancy rate and live birth rate (9). Infertile patients seeking assisted pregnancy treatment had experienced recurrent embryo implantation failures, which means higher physical and mental pressure and economic cost. In this case, clinicians need to provide patients with more reasonable clinical management strategies in time. Based on the above discussion, it’s worth discussing whether two failed treatment cycles can fully evaluate endometrial factors, so that we can take timely measures to improve the endometrium receptivity.

RIF is a complex pathological condition defined clinically, the pathogenesis is poorly revealed and mainly related to embryonic and maternal factors, such as chromosome, uterine anatomical abnormalities and maternal immune dysfunction (5, 10). In addition to being closely related to the parental karyotype, there were multiple endometrial receptivity-related genes had been found to predict the occurrence of RIF (11). The down-regulation of these genes in RIF patients affects cell regulation and division, as well as the formation of cytoskeleton and cilia. Macroscopically, uterine anatomical abnormalities affecting endometrial receptivity include polyps, myomas, adhesions, septate uterus and thin endometrium (4). Furthermore, a variety of immune factors are also important factors causing embryo implantation failure (10), and one of the factors being tested recently was thyroid autoimmunity. Thyroid autoimmunity is a typical immune disease related to RIF, which not only causes RIF through thyroid dysfunction, but also is accompanied by immune imbalance (12). In view of the rapid development of ART in recent years, hysteroscopy and genetic testing before embryo implantation have been widely carried out, which have solved many visual and solvable factors causing implantation failures, such as surgery to improve uterine anatomical abnormalities and genetic diagnosis screening embryos with normal chromosomes (13, 14). However, for 28% couples with unexplained infertility, there is no pathological abnormalities (15), our improvement measures are not available, and the effect of assisted pregnancy is not ideal.

Based on the above, we believe that it is necessary to improve the clinical management strategies of RIF populations as early as possible, because its diagnostic criteria are closely related to the clinical treatment and prognosis. Whether two failed ET cycles are enough to evaluate poor endometrial receptivity, this study mainly provides clinical data for the definition of the number of cycles with RIF. Therefore, we designed this study which could be divided into two parts. First, we investigated whether the two implantation failure could be included in the diagnostic criteria of unexplained RIF. In addition, our data also compared the outcome of assisted pregnancy for the next transfer cycle in these two definitions.

## Materials and Methods

The clinical data of 1518 patients recruited from the Center for Reproductive Medicine of the First Affiliated Hospital of Zhengzhou University during January 2016 to June 2019 were collected and analyzed. Participants were included if they were under 40 years old, had undergone at least two consecutive failed ET cycles (including fresh and frozen transferred cycles) and had failed to achieve a clinical pregnancy after transferring at least four cleavage embryos or two blastocysts. Exclusion criteria included patients with adenomyosis, endometritis, uterine anatomical abnormalities, obvious intrauterine adhesions or occupation that had not been removed, chromosomal abnormalities in both or one of the couples, PGD/PGS, autoimmune disease, oocyte or sperm donation, thyroid dysfunction, hypertension and diabetes.

ET cycles experienced by participants included fresh and frozen transferred cycles, and the protocols of ovulation induction in fresh cycles included long protocol, super long protocol, modified super long protocol; the endometrial preparation protocols in frozen transferred cycles is natural cycle and artificial cycle. For detailed programs, please refer to our center published articles (16, 17). The types of transferred embryos included cleavage embryos on the Day 3 and blastocysts on the Day 5 after fertilization. For the scoring criteria for high-quality embryos, please refer to our center published articles (18). Serum human chorionic gonadotrophin (hCG) concentrations were measured on days 14 and 18 after embryo transfer. A transabdominal ultrasound was performed on 35 days after embryo transfer to determine whether there was an intrauterine gestational sac after hCG positive pregnancy test. A positive hCG test without gestational sac was defined as biochemical pregnancy and the presence of an intrauterine gestational sac was defined as clinical pregnancy (19).

**Figure 1:**
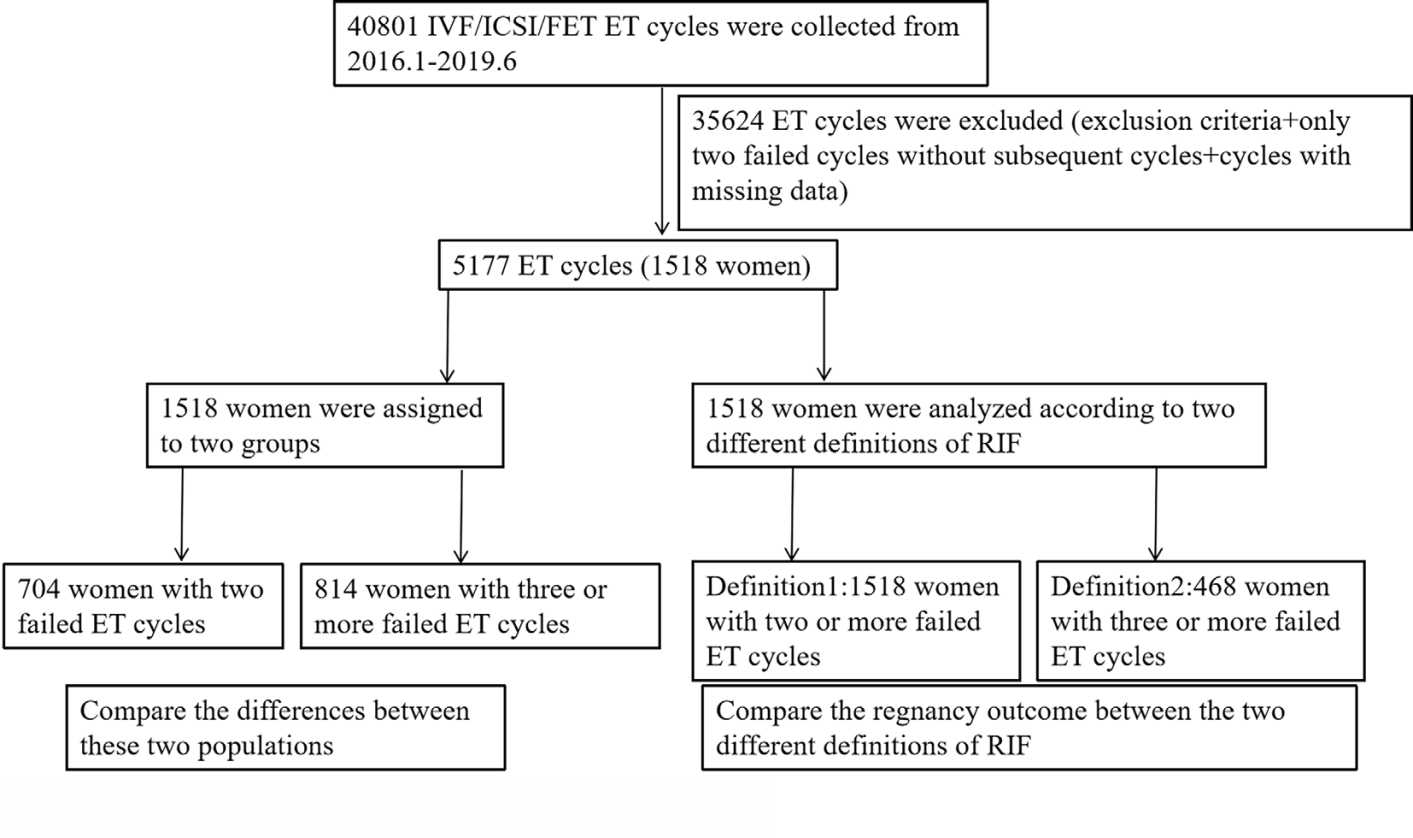
Flow chart representing the present study.

Our study was divided into two parts. In the first part, we compared the related risk factors to explore the differences between these two populations. The population with two implantation failure had a clinical pregnancy at their third ET cycle. If the two implantation failure cannot be included in the diagnostic criteria of RIF, then we analyzed the pregnancy outcomes according to these two RIF definitions. The second part was a retrospective cohort study. The research route was shown in **Figure 1**. Main comparative clinical factors included age, BMI, duration of infertility, type of infertility, type of cycle, stage of embryos development, number of transferred embryos, female factors (scarred uterus, endometriosis, tubal pathology, PCOS, pelvic adhesions) and male factors (oligoasthenospermia、varicocele and teratism of testis). Main pregnancy outcome measures included live birth rate, biochemical pregnancy rate, clinical pregnancy rate, ectopic pregnancy rate and abortion rate.

### Statistical Analysis

SPSS Statistics version 21.0 was used to perform and analyze the data. Continuous variables were described by mean ± standard deviation (Mean ± SD), and differences between groups were compared by independent-sample t-test; categorical variables were described by frequency and percentage n (%), and proportions between groups were compared by chi-square test or continuous adjusted chi-square test. Binary logistic regression was used for the adjusted *OR* (odds ratio) and 95% *CI* (confidence interval). *P* < 0.05 (two-tailed) indicated that the difference was statistically significant.

## Results

### Associate Factors of Patients With Two *vs* Three or More Implantation Failure

At initial research, 704 patients had two consecutive implantation failure and had a clinical pregnancy at their next ET cycle, 814 patients had three or more consecutive implantation failure. We compared the clinical data between the two groups, and **Table 1** showed that partial general information and distribution of associated factors of patients with two *vs* three or more implantation failure were different significantly. After adjusting for confounding factors by using binary logistic regression, the results found that there were significant differences in maternal age [*P*<0.01, *aOR*=1.054(95% *CI*:1.023-1.086)], type of cycle [*P*<0.01, *aOR*=2.040(95% *CI*:1.491-2.790)], stage of embryos development [*P*<0.01, *aOR*=0.287(95% *CI*:0.218-0.377)], number of transferred embryos [*P*<0.01, *aOR*=0.184(95% *CI*:0.137-0.246)], female factor (tubal pathology) [*P*<0.05, *aOR*=0.432(95% *CI*:0.202-0.926)] and male factor [*P*<0.01, *aOR*=1.734(95% *CI*:1.222-2.460)] between the two and three or more unexplained implantation failure (**Table 2**). These results indicated that maternal age, type of cycle, stage of embryos development, number of embryos transferred, tubal pathology and male factor were independent risk factors for these two iatrogenic situations. But whether these different factors would affect the outcome of assisted pregnancy, especially the outcome of live birth, was not clear.

**Table 1:** The comparison of general information and distribution of associated factors of patients with two *vs* three or more implantation failure.

Item	2 implantation failure	≥3 implantation failure	*t/χ^2^ value*	*P value*
Total	704	814		
Age (year)	30.43 ± 4.07	31.91 ± 4.42	-6.806	<0.001*
Duration of infertility (year)	3.77 ± 2.76	4.38 ± 3.32	-3.913	<0.001*
BMI (kg/m^2^)	22.71 ± 3.32	22.80 ± 3.01	-0.543	0.587
Percentage of primary infertility (%)	42.76 (301/704)	34.64 (282/814)	10.501	0.001*
Percentage of fresh cycle (%)	10.80 (76/704)	21.62 (176/814)	31.958	<0.001*
Percentage of blastocyst transfer cycles (%)	49.72 (350/704)	30.84 (251/814)	56.267	<0.001*
No. of embryos transferred			87.060	<0.001*
1	20.60 (145/704)	43.12 (351/814)		
2	79.40 (559/704)	56.88 (463/814)		
Scarred uterus (%)	14.91 (105/704)	21.38 (174/814)	10.505	0.001*
Endometriosis (%)	4.40 (31/704)	5.77 (47/814)	1.455	0.228
Tubal pathology (%)	3.27 (23/704)	1.47 (12/814)	5.387	0.020*
PCOS (%)	18.18 (128/704)	12.29 (100/814)	10.284	0.001*
Pelvic adhesions (%)	7.53 (53/704)	8.60 (70/814)	0.582	0.446
Male factor (%)	9.09 (64/704)	15.11 (123/814)	12.665	<0.001*

(a) BMI, body mass index; PCOS, polycystic ovary syndrome; *Represents statistically significant.

**Table 2:** The comparison of associated factors of patients with two *vs* three or more implantation failure by logistic regression analysis.

Item	*B*	*aOR*	95% *CI*	*P value*
Age (year)	0.053	1.054	1.023-1.086	0.001*
Duration of infertility (year)	0.020	1.021	0.981-1.062	0.312
Percentage of primary infertility (%)	-0.081	0.922	0.712-1.194	0.538
Percentage of fresh cycle (%)	0.713	2.040	1.491-2.790	<0.001*
Percentage of blastocyst transfer cycles (%)	-1.249	0.287	0.218-0.377	<0.001*
No. of embryos transferred				
1		1		
2	-1.693	0.184	0.137-0.246	<0.001*
Scarred uterus (%)	-0.191	0.826	0.599-1.139	0.244
Tubal pathology (%)	-0.839	0.432	0.202-0.926	0.031*
PCOS (%)	-0.120	0.887	0.645-1.218	0.457
Male factor (%)	0.550	1.734	1.222-2.460	0.002*

*Represents statistically significant.

We further analyzed the influence of these different factors on the pregnancy outcome of patients with unexplained RIF. As far as this study was concerned, the classification of patients with just two implantation failure was not clear, so we only explored the impact of these factors on the generally accepted definitions for RIF (three or more failed treatment cycles). The results were shown in **Table 3**. For patients who had three unexplained implantation failure, in the fourth cycle of IVF/ICSI/FET, the live birth rate decreased significantly with age [*P*<0.001, *aOR*=0.921(95% *CI*:0.880-0.963)], and the live birth rate of blastocyst transfer was significantly higher than that of cleavage embryo transfer [*P*=0.007, *aOR*=1.826(95% *CI*:1.180-2.825)]. But these different risk factors had no significant effect on biochemical pregnancy rates and abortion rates.

**Table 3:** The pregnancy outcome of the fourth cycle of patients with three implantation failure by logistic regression analysis.

Item	Age (year)	Type of cycle (%)		Stage of embryos development (%)		No. of embryos transferred (%)		Tubal pathology (%)	Male factor (%)
		ET	FET	Cleavage	Blastocyst	1	2		
Live birth rate									
*P*	<0.001*	0.196		0.007*		0.064		0.533	0.473
*aOR* (95% *CI*)	0.921 (0.880-0.963)	1	0.744 (0.475-1.165)	1	1.826 (1.180-2.825)	1	1.481 (0.977-2.246)	0.482 (0.049-4.773)	1.199 (0.731-1.965)
Biochemical pregnancy rate									
*P*	0.774	0.649		0.918		0.211		0.999	0.171
*aOR* (95% *CI*)	1.014 (0.922-1.116)	1	0.802 (0.310-2.074)	1	1.05 0.412-2.678)	1	0.565 (0.231-1.383)	0.000	1.908 (0.756-4.815)
Abortion rate									
*P*	0.298	0.989		0.416		0.126		0.149	0.656
*aOR* (95% *CI*)	1.047 (0.960-1.142)	1	0.994 (0.407-2.42)	1	1.431 (0.603-3.394)	1	1.957 (0.828-4.627)	5.651 (0.538-59.40)	1.238 (0.484-3.166)

For patients who had three unexplained implantation failure, in the fourth cycle of ET, live birth rate decreased significantly with age ( P < 0.001), and the live birth rate of blastocyst transfer was significantly higher than that of cleavage embryo transfer (P = 0.007). *represents statistical significance.

From the above results, we could find that maternal age and stage of embryos development were the main differential factors between the two populations, and had a significant impact on the subsequent pregnancy outcome. Thence, patients with two consecutive failed treatment cycles cannot be included in the population with three or more consecutive implantation failure.

### Comparison of Main Pregnancy Outcome Measures

**Figure 2:**
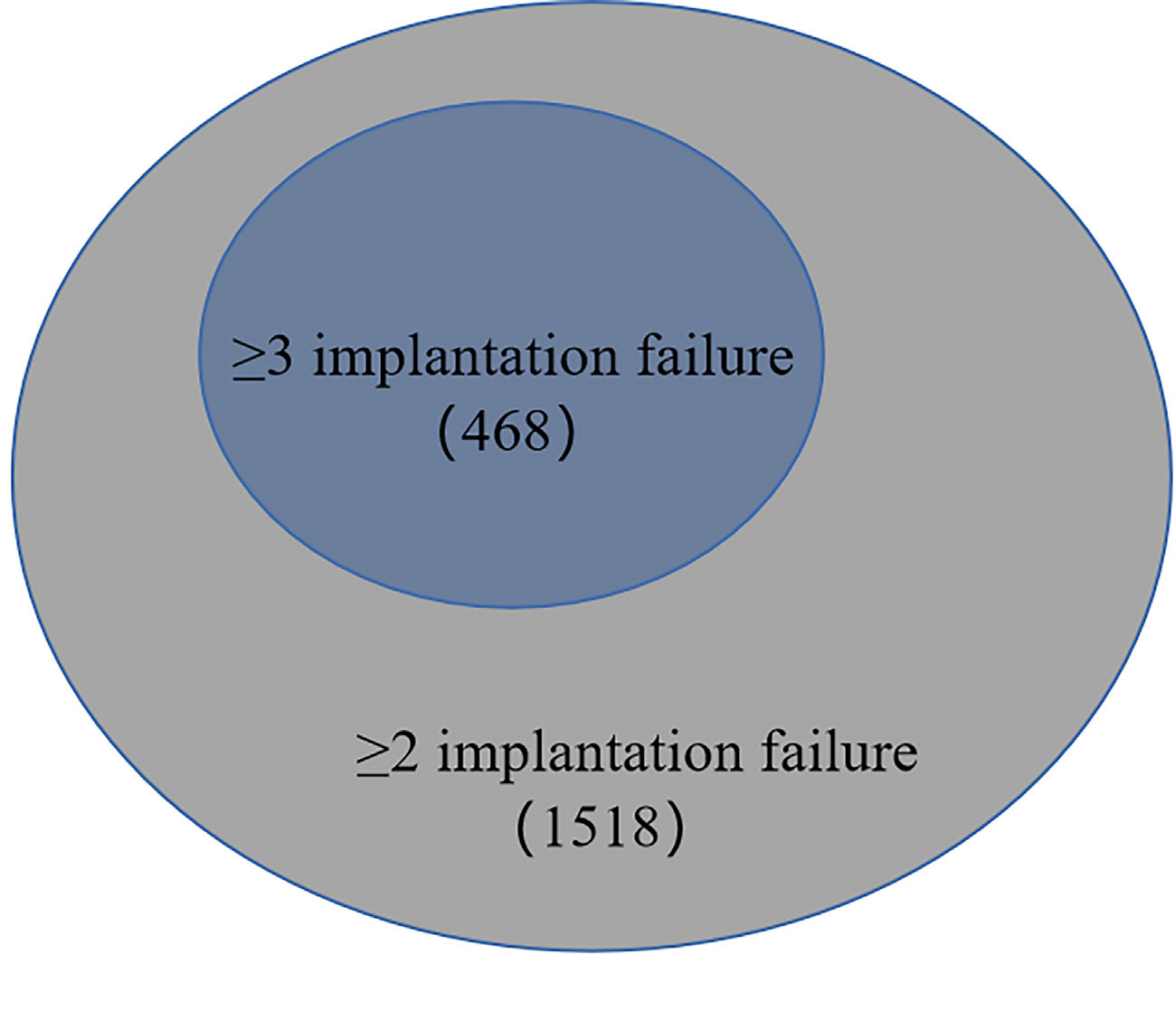
The number of RIF patients with two different definitions.

In the second part of the article, we analyzed the pregnancy outcomes of patients according to these two different definitions for RIF (**Figure 2**). Results as shown in **Table 4**, 1518 patients had at least two implantation failure, and 468 patients had at least three implantation failure with subsequent assisted pregnancy cycles. At their first assisted pregnancy treatment after the diagnosis of RIF according to different definitions, there were no significant difference in the biochemical pregnancy rate (6.39 *vs* 5.13%, *P*=0.318), clinical pregnancy rate (46.44 *vs* 50.85%, *P*=0.095), ectopic pregnancy rate (1.52 *vs* 1.28%, *P*=0.713) and abortion rate (9.29 *vs* 6.62%, *P*=0.073) between the two definitions, but the live birth rate (35.64 *vs* 42.95%, *P*=0.004) was significantly different.

**Table 4:** Comparison of main pregnancy outcome measures.

Item	≥2 implantation failure	≥3 implantation failure	*χ^2^*	*P value*
Total	1518	468		
Live birth rate (%)	35.64 (541/1518)	42.95 (201/468)	8.167	0.004*
Biochemical pregnancy rate (%)	6.39 (97/1518)	5.13 (24/468)	0.995	0.318
Clinical pregnancy rate (%)	46.44 (705/1518)	50.85 (238/468)	2.792	0.095
Ectopic pregnancy rate (%)	1.52 (23/1518)	1.28 (6/468)	0.135	0.713
Abortion rate (%)	9.29 (141/1518)	6.62 (31/468)	3.211	0.073

*Represents statistically significant.

## Discussion

In this study, we compared associated and prognostic factors in 1518 patients with two implantation failure *vs* three or more implantation failure from our Center for Reproductive Medicine during January 2016 to June 2019. And we found two implantation failure cannot be included in the commonly accepted diagnostic criteria of RIF, the patients with only two implantation failure would be considered as false-positive diagnosis of RIF.

The exact definition of RIF remains controversial, and considering the number of failed cycles, three and two consecutive failed treatment cycles are the most commonly used threshold (3). Recently, theoretical model in some studies suggested that inappropriate number of failed cycles might expose patients to over-diagnosis and over-treatment (20). At the same time, due to the excellent embryo quality, the diagnosis of RIF with fewer failed cycles seems to be a more timely reflection of the endometrial receptivity (8). However, based on the results of this study, it seemed that it was inappropriate to include two implantation failure in the RIF definition. The date of RIF diagnosis was determined, defined as the first day of the menstrual period after the last failed IVF/ICSI/FET treatment (21). So, if a patient was under 40 years old and had failed to achieve a clinical pregnancy after transferring at least four cleavage embryos or two blastocysts, we could classify her at the second or third failed ET cycles (the hypothetical RIF definition: at least two failed ET cycles *vs* generally accepted RIF definition: at least three failed ET cycles). Therefore, the basic information we included was from the second failure cycle and the third failure cycle respectively.

Compared with three or more implantation failure, patients with just two implantation failure were significantly younger. There was a significant statistical significance between these two populations, although their average age was relatively low. But the other side of this results suggested that women’s fertility was damaged seriously and we should pay more attention to the biological age. A recent study of 118 women who had experienced RIF showed that the median pregnancy time was just 9 months after the diagnosis of RIF (21). And for RIF patients, the live birth rate decreased significantly with age. Age is an independent risk factor for RIF patients, and will significantly affect the subsequent assisted pregnancy outcome. Female advanced age not only leads to a decrease in the number and quality of embryos, but also increases the asynchrony of embryo and endometrial development (22, 23). On the other hand, the proportion of blastocyst transfer was different between the two populations. When discussing associated factors and prognosis, in agreement with previous reports, results showed that blastocyst transfer was a preferred strategy than cleavage embryo transfer (24).

A uniform definition is important for standardizing research protocols and adopting a uniform approach to patients with RIF in scientific research. These stressed couples who are frequently overwhelmed by unsuccessful childbearing should be given more gentle, caring, caring early intervention. When we tried to incorporate two implantation failure into diagnostic criteria of RIF, it could be seen that patients with only two implantation failure would be considered as false-positive diagnosis of RIF, accompanied by an abnormally lower live birth rate. We collected clinical data of 1518 patients with two or more failed ET cycles. As shown in **Figures 1**, **2**, when we adopted two or more implantation failure to define RIF, 1518 patients could be included in the RIF population and the live birth rate of RIF patients in the first pregnancy outcome after diagnosis was (541/1518) 35.64%. In the same way, when we adopted three or more implantation failure to define RIF, just 468 patients could be included in the RIF population, and the live birth rate of RIF patients in the first pregnancy outcome after diagnosis was (201/468) 42.95%. This was because When we calculated the live birth rate under the first definition (two or more implantation failure), its denominator excessively increased the number of third failed ET cycles (468 patients). And when we calculated the live birth rate under the second definition (three or more implantation failure), its denominator excessively decreased the number of these two conditions: 1) only two implantation failure and had a clinical pregnancy at their third ET cycle (704 patients); 2) just three implantation failure without subsequent cycle (346 patients). The most important was that above calculation of living rate was based on dividing patients into RIF population, that was, the living rate of patients in RIF population, rather than in the overall infertility population.

We needed to emphasize two key points again: 1) the living rate could only represent the pregnancy outcome of patients in their respective RIF defined population, rather than in the overall infertility population; 2) according to the first part of our article, the patients with only two implantation failure could not be classified into RIF population, which would be considered as false-positive diagnosis of RIF, accompanied by an abnormally lower live birth rate. This phenomenon would lead to a pressures of clinicians and patients and inappropriate clinical management strategies. And in this latest study, they adopted the threshold definition was the majority view based on the three failed treatment cycles (2). Furthermore, it is worth noting that with the advancement of ART, patient characteristics are constantly changing, we should keep exploring the most appropriate definition, physicians should not irrationally comply with these requests.

The diagnosis of RIF is a challenging and frustrating condition world-wide, but the population included in this study only includes Chinese, so the main limitation in our study is regional and ethnic differences. Moreover, more and more clinicians will incorporate lifestyles such as smoking, drinking, drugs in consideration of RIF, and this study is less concerned about this (2). As a retrospective analysis, although we have tried our best to eliminate various biases, the existence of inherent biases may still affect the results of the study, and more and larger samples of clinical and basic research are needed for verification.

## Conclusion

This study assessed the couples with two *vs* three or more implantation failure regarding the discussion of defining RIF. And two consecutive failed treatment cycles cannot be included in the diagnostic criteria of RIF. This study supports the generally accepted definition of three or more failed treatment cycles for RIF.

## Data Availability Statement

The raw data supporting the conclusions of this article will be made available by the authors, without undue reservation.

## Ethics Statement

The studies involving human participants were reviewed and approved by Institutional Review Board (IRB) of First Affiliated Hospital of Zhengzhou University. According to the principles of Good Clinical Practice and the Declaration of Helsinki, various parameters kept anonymized records, with no access to identifying information. Written informed consent for participation was not required for this study.

## Author Contributions

YyS conducted the analysis and wrote the manuscript. YZ and XM collected the data. WJ statistically analyzed the data. YcS conceived the study design, contributed to interpretation of the data, critically revised the article, and approved the final version. All authors contributed to the article and approved the submitted version.

## Acknowledgments

We thank Yaping Liu and Xiaoli Chen for their help in recording data. We thank staffs and patients in the Center for Reproductive Medicine of the First Affiliated Hospital of Zhengzhou University for research support. 

## Conflict of Interest

The authors declare that the research was conducted in the absence of any commercial or financial relationships that could be construed as a potential conflict of interest.

## References

[1] Coughlan C Ledger W Wang Q Liu FH Demirol A Gurgan T. Recurrent Implantation Failure: Definition and Management. Reprod BioMed Online (2014) 28:14–38. 10.1016/j.rbmo.2013.08.011 24269084

[2] Cimadomo D Craciunas L Vermeulen N Vomstein K Toth B. Definition, Diagnostic and Therapeutic Options in Recurrent Implantation Failure: An International Survey of Clinicians and Embryologists. Hum Reprod (2021) 36:305–17. 10.1093/humrep/deaa317 33313697

[3] Polanski LT Baumgarten MN Quenby S Brosens J Campbell BK Raine-Fenning NJ. What Exactly do We Mean by ‘Recurrent Implantation Failure’? A Systematic Review and Opinion. Reprod BioMed Online (2014) 28:409–23. 10.1016/j.rbmo.2013.12.006 24581986

[4] Bashiri A Halper KI Orvieto R. Recurrent Implantation Failure-Update Overview on Etiology, Diagnosis, Treatment and Future Directions. Reprod Biol Endocrinol (2018) 16:121. 10.1186/s12958-018-0414-2 30518389 PMC6282265

[5] Cakiroglu Y Tiras B. Determining Diagnostic Criteria and Cause of Recurrent Implantation Failure. Curr Opin Obstet Gynecol (2020) 32:198–204. 10.1097/GCO.0000000000000620 32251092

[6] Xie H Zeng H He D Liu N. Effect of Intrauterine Perfusion of Human Chorionic Gonadotropin Before Embryo Transfer After Two or More Implantation Failures: A Systematic Review and Meta-Analysis. Eur J Obstet Gynecol Reprod Biol (2019) 243:133–8. 10.1016/j.ejogrb.2019.10.039 31704529

[7] Cao H You D Yuan M Xi M. Hysteroscopy After Repeated Implantation Failure of Assisted Reproductive Technology: A Meta-Analysis. J Obstet Gynaecol Res (2018) 44:365–73. 10.1111/jog.13571 29297956

[8] Sebastian-Leon P Garrido N Remohí J Pellicer A Diaz-Gimeno P. Asynchronous and Pathological Windows of Implantation: Two Causes of Recurrent Implantation Failure. Hum Reprod (2018) 33:626–35. 10.1093/humrep/dey023 29452422

[9] Steiner N Shrem G Tannus S Dahan SY Balayla J Volodarsky-Perel A. Effect of GnRH Agonist and Letrozole Treatment in Women With Recurrent Implantation Failure. Fertil Steril (2019) 112:98–104. 10.1016/j.fertnstert.2019.03.021 31133384

[10] Vomstein K Voss P Molnar K Ainsworth A Daniel V Strowitzki T. Two of a Kind? Immunological and Clinical Risk Factors Differ Between Recurrent Implantation Failure and Recurrent Miscarriage. J Reprod Immunol (2020) 141:103166. 10.1016/j.jri.2020.103166 32623188

[11] Koot YE van Hooff SR Boomsma CM van Leenen D Groot Koerkamp MJ Goddijn M. An Endometrial Gene Expression Signature Accurately Predicts Recurrent Implantation Failure After IVF. Sci Rep (2016) 6:19411. 10.1038/srep19411 26797113 PMC4726345

[12] Huang C Liang P Diao L Liu C Chen X Li G. Thyroid Autoimmunity Is Associated With Decreased Cytotoxicity T Cells in Women With Repeated Implantation Failure. Int J Environ Res Public Health (2015) 12:10352–61. 10.3390/ijerph120910352 PMC4586615 26308040

[13] Mao X Wu L Chen Q Kuang Y Zhang S. Effect of Hysteroscopy Before Starting In-Vitro Fertilization for Women With Recurrent Implantation Failure: A Meta-Analysis and Systematic Review. Med (Baltimore) (2019) 98:e14075. 10.1097/MD.0000000000014075 PMC6408091 30762725

[14] Greco E Bono S Ruberti A Lobascio AM Greco P Biricik A. Comparative Genomic Hybridization Selection of Blastocysts for Repeated Implantation Failure Treatment: A Pilot Study. BioMed Res Int (2014) 2014:457913. 10.1155/2014/457913 24779011 PMC3980987

[15] Expert Panel on Women’s Imaging DJ W Reinhold C EA A SM A OR B. ACR Appropriateness Criteria^®^ Female Infertility. J Am Coll Radiol (2020) 17:S113–24. 10.1016/j.jacr.2020.01.018 32370955

[16] Hu L Bu Z Guo Y Su Y Zhai J Sun Y. Comparison of Different Ovarian Hyperstimulation Protocols Efficacy in Poor Ovarian Responders According to the Bologna Criteria. Int J Clin Exp Med (2014) 7:1128–34. PMC4057873 24955194

[17] Bu Z Wang K Dai W Sun Y. Endometrial Thickness Significantly Affects Clinical Pregnancy and Live Birth Rates in Frozen-Thawed Embryo Transfer Cycles. Gynecol Endocrinol (2016) 32:524–8. 10.3109/09513590.2015.1136616 26942778

[18] Jin HX Dai SJ Song WY Yao GD Shi SL Sun YP. Embryo Developmental Potential of Microsurgically Corrected Human Three-Pronuclear Zygotes. Syst Biol Reprod Med (2015) 61:96–102. 10.3109/19396368.2014.986693 25411094

[19] Zeadna A Son WY Moon JH Dahan MH. A Comparison of Biochemical Pregnancy Rates Between Women Who Underwent IVF and Fertile Controls Who Conceived Spontaneously†. Hum Reprod (2015) 30:783–8. 10.1093/humrep/dev024 25678573

[20] Somigliana E Vigano P Busnelli A Paffoni A Vegetti W Vercellini P. Repeated Implantation Failure at the Crossroad Between Statistics, Clinics and Over-Diagnosis. Reprod BioMed Online (2018) 36:32–8. 10.1016/j.rbmo.2017.09.012 29102484

[21] Koot YEM Hviid SM Goddijn M de Bever S Eijkemans MJC Wely MV. What Is the Prognosis for a Live Birth After Unexplained Recurrent Implantation Failure Following IVF/ICSI? Hum Reprod (2019) 34:2044–52. 10.1093/humrep/dez120 31621857

[22] Forman EJ Upham KM Cheng M Zhao T Hong KH Treff NR. Comprehensive Chromosome Screening Alters Traditional Morphology-Based Embryo Selection: A Prospective Study of 100 Consecutive Cycles of Planned Fresh Euploid Blastocyst Transfer. Fertil Steril (2013) 100:718–24. 10.1016/j.fertnstert.2013.04.043 23725804

[23] Shapiro BS Daneshmand ST Desai J Garner FC Aguirre M Hudson C. The Risk of Embryo-Endometrium Asynchrony Increases With Maternal Age After Ovarian Stimulation and IVF. Reprod BioMed Online (2016) 33:50–5. 10.1016/j.rbmo.2016.04.008 27178763

[24] Reljič M Knez J Kovač V Kovačič B. Endometrial Injury, the Quality of Embryos, and Blastocyst Transfer Are the Most Important Prognostic Factors for In Vitro Fertilization Success After Previous Repeated Unsuccessful Attempts. J Assist Reprod Genet (2017) 34:775–9. 10.1007/s10815-017-0916-4 PMC5445053 28386815

